# Supplementary material for: Human Brain Endothelial Cell-Derived Extracellular Vesicles Reduce Toxoplasma gondii Infection In Vitro in Human Brain and Umbilical Cord Vein Endothelial Cells
Source: Int J Mol Sci. 2025 Mar 14;26(6):2640. doi: 10.3390/ijms26062640 (PMC11942338; doi:10.3390/ijms26062640)
Supplement: Supplementary file 1 [file ijms-26-02640-s001.zip › ijms-3447240-supplementary.pdf]

Title: *Human brain endothelial cell-derived extracellular vesicles reduce Toxoplasma gondii infection in vitro in human brain and umbilical cord vein endothelial cells*

Authors: Luiz Fernando Cardoso Garcia[1]; Victoria Cruz Cavalari[1]; Pryscilla Fanini Wowk[2], Letusa Albrecht[1,*].

Affiliation: 1: Laboratório de Pesquisa em Apicomplexa – Instituto Carlos Chagas, FIOCRUZ-PR. 2: Grupo de Pesquisa em Imunologia Molecular, Celular e Inteligência Artificial – Instituto Carlos Chagas, FIOCRUZ-PR.

***:** corresponding author.

SUPPLEMENTARY DATA AND SUBTITLES

Supplementary Table S1. Transcriptional changes in HBMEC and HUVEC in infection with *T. gondii* are distinct.

	HBMEC					HUVEC				
	**Hour 0**	**Hour 1**	**Hour 2**	**Hour 4**	***p*-value**	**Hour 0**	**Hour 1**	**Hour 2**	**Hour 4**	***p*-value**
ADAMTS13	1	0.39	0.53	0.00	<0.0001	1	0.95	0.00	0.00	<0.0001
ANG1	0	0.00	0.00	0.00	0	1	1.04	1.35	0.97	0.6772
ANG2	0	0.00	0.00	0.00	0	1	1.00	0.67	0.49	0.4146
ENOS	1	0.65	0.62	0.97	0.0476	1	1.00	0.67	0.49	0.5835
ICAM1	1	1.05	0.00	0.00	<0.0001	1	1.80	1.04	0.85	0.3887
IL6	1	0.93	1.06	1.06	0.829	1	0.94	2.80	3.83	0.0006
IL8	1	1.63	3.20	2.85	0.0722	1	1.63	3.19	5.57	0.0011
MCP1	1	0.87	2.40	0.00	0.3186	1	3.76	17.82	18.74	0.0002
SDC1	1	0.91	1.19	0.95	0.2443	1	0.20	1.04	0.73	0.3141
SELECTINP	0	0.00	0.00	0.00	0	1	1.35	0.89	1.88	0.4395
SOCS3	1	1.18	1.17	1.33	0.7215	1	1.42	3.59	2.17	0.0025
VCAM1	0	0.00	0.00	0.00	0	1	1.38	3.57	7.50	<0.0001
VEGF	1	1.19	1.18	1.00	0.1825	1	0.68	2.12	1.98	0.0162

HBMEC and HUVEC were infected with *T. gondii* and incubated for 1, 2, or 4 hours. Next, the RNA from the cells was extracted, converted to cDNA, and the transcription pattern was evaluated by qPCR and presented in a table. *p*-value indicates statistical differences in the comparison between time points for each cell model. Data were obtained from three biological and technical replicates and are expressed as the mean. ANOVA/Tukey.

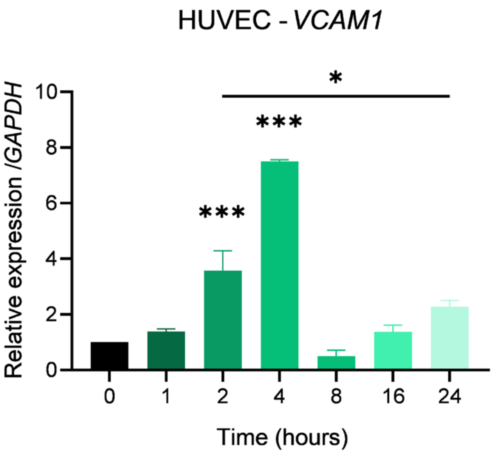

Supplementary Figure S1. Transcriptional change of *VCAM1* in HUVEC. HUVEC were infected by *T. gondii* and waited 1, 2, 4, 8, 16 and 24 hours. Next, the RNA from the cells was extracted, converted to cDNA and the transcription pattern was evaluated by qPCR and presented in the form of a bar graph. Analysis of uninfected cells was considered as time 0. Data were obtained from three biological and technical replicates and are expressed as the mean ± standard deviation. For better visualization, only a few statistically significant differences were presented. ANOVA/Tukey. *: p-value < 0.05; ***: p-value < 0.001.

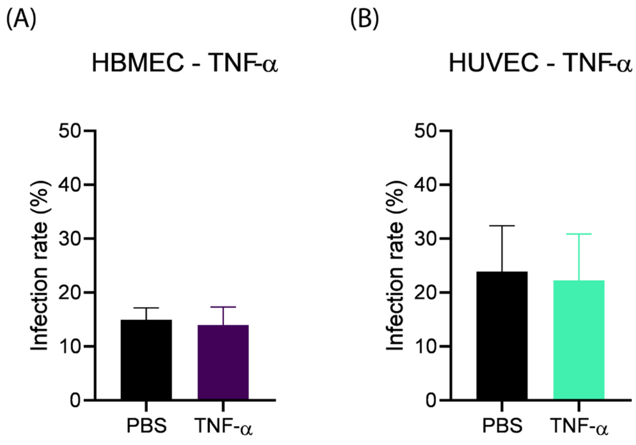

Supplementary Figure S2. TNF-α does not promote a protective effect on HBMEC and HUVEC infection by *T. gondii*. About 6.4×10^3 HBMECs and HUVEC were cultured in 96-well plate with 100 µL of DMEM and EBM™-2. Then, they were incubated for 24 hours with TNF-α (10 mg/mL) or with the addition of 1 uL of PBS. After incubation, cells were infected at MOI-5 with *T. gondii*, strain ME-49 and, after one hour of infection, the cells were washed with PBS, fixed with 4% paraformaldehyde, labeled with DAPI and Evans Blue and the infection rate was evaluated by fluorescence microscopy. All cells in three different fields per experimental unit were counted. Data obtained from three biological replicates and expressed as the mean ± standard deviation. Simple t-test (**A**, **B**).

Supplementary Table S2. EV markers found by LC-MS/MS.

	**Mean Intensity**

	HB-EV	HBTg-EV	Tg-EV	HU-EV	HUTg-EV
Annexin A2	154050	1165900	2765600	30618000	56028000
CD44	0	75210	149770	669410	1002600
HSPA7	0	0	132840	668500	803300

EVs obtained by differential centrifugation from 1.0×10^9 *T. gondii* tachyzoites, HUVEC or HBMEC infected or not by *T. gondii*. The samples were analyzed by LC-MS/MS. Reference proteomes for *Homo sapiens* and *T. gondii* were acquired from the UniProt database and columns represented by the mean intensity of label free quantification (LFQ Intensity) for each sample.

Supplementary Table S3. Sequence of primers selected for the assessment of endothelial activation.

Gene	Fragment (pb)	Sequence
ACTINB	224	F: 5'- AGGATGCAGAAGGAGATCACT -3'
		F: 5'- GGGTGTAACGCAACTAAGTCATAG -3'
ADAMTS13	84	F: 5'- CACAGGCCTCTCTTCACACA -3'
		R: 5'- GGTGTTAGGGGAGATGCTCA -3'
ANG1	99	F: 5'- CAATGGGGGAGGTTGGACTGTA -3'
		R: 5'- GAGGGATTTCCAAAACCCATTTTAT -3'
ANG2	69	F: 5'- ACGTGAGGATGGCAGCGTT -3'
		R: 5'- GAAGGGTTACCAAATCCCACTTTAT -3'
ENOS	235	F: 5'- TGCTGGCATACAGGACTCAG -3'
		R: 5'- AGCCCTTTGCTCTCAATGTC -3'
GAPDH	99	F: 5'- GGCCTCCAAGGAGTAAGACC -3'
		R: 5'- GACTGAGTGTGGCAGGGACT -3'
ICAM1	217	F: 5'- GGCCTCAGTCAGTGTGA -3'
		R: 5'- AACCCCATTCAGCGTCA -3'
IL6	149	F: 5'- ACTCACCTCTTCAGAACGAATTG -3'
		R: 5'- CCATCTTTGGAAGGTTCAGGTTG -3'

IL8	229	F: 5’- TCTGCAGCTCTGTGTGAAGG -3’<br>R: 5’- ACTTCTCCACAACCCTCTGC -3’
SDC1	71	F: 5’- AGGACGAAGGCAGCTACTCCT -3’<br>R: 5’- TTTGGTGGGCTTCTGGTAGG -3’
SELECTINP	130	F: 5’- CTGTTACCCTGGATTCTATGGGC -3’<br>R: 5’- GCTGCACTGCGAGTTAAAAGAG -3’
SOCS3	109	F: 5’- GGCCACTCTTCAGCATCTC -3’<br>R: 5’- ATCGTACTGGTCCAGGAACTC -3’
TNF	272	F: 5’- CTG GGCAGGTCTACTTTGGG -3’<br>R: 5’- CTGGAGGCCCCAGTTTGAAT -3’
VCAM1	132	F: 5’- AGTTGAAGGATGCGGGAGTA -3’<br>R: 5’- AGAGCACGAGAAGCTCAGGA -3’
VEGF	81	F: 5’- TGCAGATTATGCGGATCAAACC -3’<br>R: 5’- TGCATTCACATTTGTTGTGCTGTAG -3’
VWF	305	F: 5'-TCGGACCCTTATGACTTTGC-3'<br>R: 5'-TACAGCACCATTCCCTCCTG-3'

Sequence of primers selected for the assessment of endothelial activation. The table includes the name of the gene to be evaluated, the approximate size of the amplicon generated by qPCR in base pairs (bp) and the sequence of the primers.
